# The Predictive Role of Chinese EFL Teachers’ Individual Self-Efficacy and Collective Efficacy in Their Work Engagement

**DOI:** 10.3389/fpsyg.2021.752041

**Published:** 2021-09-16

**Authors:** Jianbo Yang

**Affiliations:** Center for Second Language Writing Research/School of College English Teaching and Research, Henan University, Kaifeng, China

**Keywords:** self-efficacy, collective efficacy, work engagement, foreign language learning, Chinese EFL teachers

## Abstract

Both teacher individual self-efficacy (TSE) and collective efficacy (TCE) are indispensable since they impact the amount to which teachers are engaged in their work. Although several studies have been carried out considering the referred points, it seems to be a must to conduct such studies among Chinese teachers to measure the link between these three variables. In this study, the author has made endeavors to scrutinize the interplay among TSE, TCE, and work engagement (WE), and also the significant role of self-efficacy and collective efficacy in WE. Participants were 346 university professors and middle school teachers, from 25 provinces of China. Results substantiate that TSE and TCE predict teachers’ WE. In other words, the more efficacious a teacher is, the more he is engaged in his work. In the discussion part, the consistency between the current study and some other studies with the same topic is unpacked. Then, some limitations are discussed and further implications are suggested.

## Introduction

In the realm of teaching and learning, teachers have become the center of attention since when the teachers feel emotionally balanced and well-adjusted, the students will be positively affected, and it incredibly facilitates the process of learning, which is the reason behind this research. Nevertheless, the teacher-student interrelationships have received due attention on the part of researchers; however, surprisingly little attention has been focused on teachers’ intrapersonal relationships. Regarding the above-mentioned points, teacher stress and teacher self-efficacy (TSE) have been said to predict the amount of engagement, emotional boredom, and the amount of motivation when leaving the profession of teaching (Skaalvik and Skaalvik, 2016). It was also revealed that the students’ desirable academic outcomes can be facilitated by positive teacher interpersonal communication behaviors (Xie and Derakhshan, 2021). It was also indicated that TSE and teacher collective efficacy (TCE) are positively linked to teachers’ wellbeing (Fathi et al., 2020).

In order to tackle this issue, studies should have been conducted to address related variables relevant to teachers themselves, since their pivotal role in the learning context is not negligible (Jex and Bliese, 1999). Therefore, considering this point, this study was carried out to unravel the relationship among three aspects of teachers’ variables that are found to be efficacious in teachers’ WE.

The central aim of the present study is to conduct more research on the paramount effect of Chinese EFL TSE and TCE in their WE. First of all, the following terms “teacher self-efficacy” and “collective self-efficacy” have been defined. Then, WE with its subfactors has been introduced. The methodology of the current study is what has been explained next. The results shown in different tables were discussed. In the end, the limitations of the study and further implications have been suggested. Steps have been taken to answer the following questions in this research:

1. Is there any link between Chinese EFL teachers’ self-efficacy, collective efficacy, and their work engagement?

2. How can teachers’ work engagement be anticipated by Chinese EFL teachers’ self-efficacy and collective efficacy?

## Literature Review

### Teacher Individual Self-Efficacy and Collective Self-Efficacy

It was emphasized that TSE refers to the extent to which a teacher believes in his personal abilities which affect students’ outcomes (Wheatley, 2005) although the conceptualization of this term differs from researcher to researcher (Skaalvik and Skaalvik, 2007). The theoretical postulations of TSE have emanated from two orientations. Rotter’s (1966) model highlighted the role of internal and external control; it has been said that teachers take the view that if students’ accomplishments and behavior are positively impacted by education, their TSE dramatically increases (Guskey and Passaro, 1994). TSE has also been supposed to reduce if teachers are of the belief that external teaching factors, such as students’ capabilities and family background, are of great importance in terms of students’ learning a new language rather than the impact the teachers have (Goddard and Goddard, 2001). In the second model as opposed to this concept, it is highlighted by Bandura (1997) that self-efficacy, formally known as TCE is conceptualized as what teachers hold about their abilities to organize and conduct tasks, needed to reach given educational goals. In this regard, people are inclined to do the tasks that they think they have the capability to control, and in contrast, they stop doing the tasks that they suppose are beyond their abilities (Schwarzer, 1999; Garrido, 2000). It is not just teachers’ WE but students’ engagement is impacted by TSE and some other variables, like teachers’ credibility, stroke, and success (Pishghadam et al., 2021; Wang et al., 2021). It has also been mentioned that there is a positive and significant link between collective teacher efficacy and the amount of teachers’ commitment to students (Lee et al., 2011).

### Work Engagement

Engagement is described as a steady conative-affective state rather than a transient state. WE comprises three sub-constructs, vigor which is characterized by how energetic and resilient a teacher is as working, the willingness to make attempts that is invested in working, and consistency when facing difficulties. Dedication is the second sub-construct of WE which refers to the level of involvement while working and gaining experience. The third sub-component of WE is absorption that refers to the full concentration and immersion when working (Maslach et al., 2001).

### The Impacts of Teacher Self-Efficacy and Collective Efficacy on Students’ Engagement

In an effort to illustrate the significance of TSE and TCE, it has been noted that efficacious teachers who trust in their own and their group’s professional capacities have more inclination to implement new instructional methods and approaches which urge the students to take part in classroom activities (Papa, 2015). It has also been explicated that efficacious teachers positively affect the students which leads to them being more engaged in the classroom (Salanova et al., 2011). It has also been proposed that efficacious teachers commonly exhibit higher consistency and attempt, by which students are inspired to become engaged in the learning process (Tschannen-Moran and Barr, 2004).

## Materials and Methods

### Participants

In this study, the final 346 participants were from more than 50 cities from 25 provinces of China. As it is unraveled in Table 1, out of 25 provinces of China from which participants took part, 223 teachers were from Henan, Zhejiang, Tianjin, Ningxia, Inner Mongolia, Hunan, and Guangxi were among the provinces with just one participant. Most of them are university professors, and they were heterogeneous in terms of gender, with 69 male teachers and 277 female teachers, and teaching experience, between 1year of teaching experience and above 25years, and also age, ranging from 32 to 62. They were opted for based on random sampling.

**Table 1:** Demographic information of the participants from each region.

Anhui	2
Beijing	12
Chongqing	6
Guangdong	2
Guangxi	1
Guizhou	10
Hainan	2
Hebei	2
Heilongjiang	2
Henan	223
Hubei	2
Hunan	1
Inner Mongolia	1
Jiangsu	12
Jilin	2
Liaoning	7
Ningxia	1
Shandong	5
Shanghai	4
Shanxi(山西)	37
Shanxi(陕西)	3
Tianjin	1
Xinjiang	2
Yunnan	5
Zhejiang	1

### Data Collection Procedure

The revised questionnaire consists of four sections and 54 items in total. To increase the credibility of the data, we translated the items into Chinese since the study is about Chinese EFL teachers and a questionnaire in the Chinese language seems friendlier to them. The Chinese version items were put into *Wenjuanxing*, a computer program for conducting an online survey in China, and an E-version questionnaire was generated.

The author sent the link of the questionnaire to English teachers *via* WeChat message, posted the questionnaire on WeChat moments, and extended an invitation to participate in WeChat groups. It began on July 22 and lasted for 5days.

### Instruments

TCE was evaluated by the scale designed by Skaalvik and Skaalvik (2007). The scale consists of seven items and is a unidimensional scale. The items were concerned with motivation, instruction, addressing students’ needs, controlling student behavior, and establishing a secure atmosphere. “Efficacy for instructional strategies, efficacy for classroom management, and efficacy for student engagement” are the sub-constructs of this conception. In previous studies, reliabilities for the full scale were from 0.92 to 0.95, and for the subscales from 0.86 to 0.90. A sample item is as follows: “As teachers of this school we can get even the most difficult students engaged in their schoolwork.” Each item was a 5-point scale ranging from false (1) to true (5).

The Teachers’ Sense of Efficacy Scale (TSES) designed and validated by Tschannen-Moran and Woolfolk Hoy (2001) was employed to measure EFL teachers’ sense of efficacy. TSES is one of the most frequently used scales measuring teachers’ sense of efficacy. It has been reported to enjoy acceptable levels of reliability and validity (e.g., Klassen et al., 2009). This scale includes 24 items. Response options ranged from 1 (nothing) to 5 (a great deal). The item examples were from (1) “How much you can do to get through to the most difficult students?” to (24) “How well can you provide appropriate challenges for competent students” (Schaufeli et al., 2006).

The original Utrecht Work Engagement Scale with 17 items designed by Schaufeli et al. (2006) was also employed to estimate how much a teacher is engaged in his job. The frequency of all items was measured on a seven-point rating scale, ranging from 0 (never) to 6 (always). A sample item is as follows: “At my job, I am very resilient, mentally.”

## Results

For the normality of data distribution to be checked, the test of Kolmogorov-Smirnov (KS) was utilized. The consequences of the normality test are indicated in Table 2.

**Table 2:** The results of KS test.

	Kolmogorov-Smirnov		
	Statistics	*df*	Sig.
Work engagement	0.06	346	0.11
Self-efficacy	0.09	346	0.06
Collective efficacy	0.07	346	0.09

The KS test results demonstrated that there is a normal distribution across all variables and parametric statistics can be utilized. Table 2 displays descriptive statistics of Chinese EFL teachers’ WE, TSE, and TCE, including the number of participants, the mean, and the standard deviation.

As Table 3 shows, 346 teachers participated in this study. Besides, it was identified that work engagement has a mean score of 90.10, TSE has a mean score of 89.54, and teachers’ collective efficacy has a mean score of 26.35. Table 4 concludes the information gathered from Cronbach alpha analyses.

**Table 3:** Descriptive statistics of the TSE, TCE, and WE.

	*N*	Minimum	Maximum	Mean	*SD*
Work engagement	346	17	119	90.10	11.06
Self-efficacy	346	27	120	89.54	10.34
Collective efficacy	346	7	35	26.35	4.77

**Table 4:** Results of Cronbach alpha indexes.

*Scale*	*Subscales*	*Cronbach alpha*
Collective efficacy	–	0.89
Work engagement	Vigor	0.87
	Dedication	0.91
	Absorption	0.88
	Overall work engagement	0.95
	Efficacy for instructional strategies	0.89
Self-efficacy	Efficacy for classroom management	0.91
	Efficacy for student engagement	0.88
	Overall self-efficacy	0.94

As can be perceived, the employed questionnaires reached acceptable indexes of Cronbach alpha as a whole in addition to their subscales.

1. Is there any relationship between Chinese EFL teachers’ self-efficacy, collective efficacy, and their work engagement?

To reply to the first research question, Pearson Correlation was employed. Table 5 demonstrates the consequences of Pearson Correlation between overall EFL teachers’ self-efficacy, collective efficacy, and their work engagement.

**Table 5:** Results of Pearson Correlation between overall EFL teachers’ self-efficacy, collective efficacy, and their work engagement.

		Work engagement	Self-efficacy	Collective efficacy
Work engagement	Pearson Correlation	1		
	Sig. (two-tailed)			
	*N*	346		
Self-efficacy	Pearson Correlation	0.58^**^	1	
	Sig. (two-tailed)	0.000		
	*N*	346	346	
Collective efficacy	Pearson Correlation	0.52^**^	0.68^**^	1
	Sig. (two-tailed)	0.000	0.000	
	*N*	346	346	346

** Correlation is significant at the 0.01 level (two-tailed).

Table 5 presents that there is a positive huge connection between teachers’ WE and TSE (*r*=0.58, *n*=346, *p*=0.000, *α*=0.01) and their collective efficacy (*r*=0.52, *n*=346, *p*=0.000, *α*=0.01). Moreover, overall teacher self-efficacy and their collective efficacy are positively linked (*r*=0.68, *n*=346, *p*=0.000, *α*=0.01).

Table 6 demonstrates the consequences of Pearson Correlation between all sub-constructs WE and overall collective efficacy.

**Table 6:** Results of Pearson Correlation between all sub-constructs work engagement and overall collective efficacy.

	Vigor	Dedication	Absorption
Collective efficacy	0.50^**^	0.53^**^	0.48^**^

** Correlation is significant at the 0.01 level (two-tailed).

As Table 6 demonstrates, there are positive significant relationships between all sub-constructs WE and overall TCE: vigor (*r*=0.50, *n*=346, *p*=0.000, *α*=0.01), dedication (*r*=0.53, *n*=346, *p*=0.000, *α*=0.01), and absorption (*r*=0.48, *n*=346, *p*=0.000, *α*=0.01).

Table 7 delineates the results of Pearson Correlation between all sub-components of WE and all sub-components of TSE.

**Table 7:** Results of Pearson Correlation between all sub-constructs work engagement and all sub-constructs of TSE.

	Vigor	Dedication	Absorption
Instructional strategies	0.56^**^	0.55^**^	0.53^**^
Classroom management	0.45^**^	0.49^**^	0.46^**^
Student engagement	0.53^**^	0.50^**^	0.49^**^

** Correlation is significant at the 0.01 level (two-tailed).

As Table 7 demonstrates, all WE sub-constructs and all self-efficacy sub-constructs are positively correlated: Among these variables, the highest relationship is ascribed to instructional strategies and vigor (*r*=0.56, *n*=346, *p*=0.000, *α*=0.01), classroom management has the highest relationship with dedication (*r*=0.49, *n*=346, *p*=0.000, *α*=0.01), and student engagement has the highest relationship with vigor (*r*=0.53, *n*=346, *p*=0.000, *α*=0.01).

**Figure 1:**
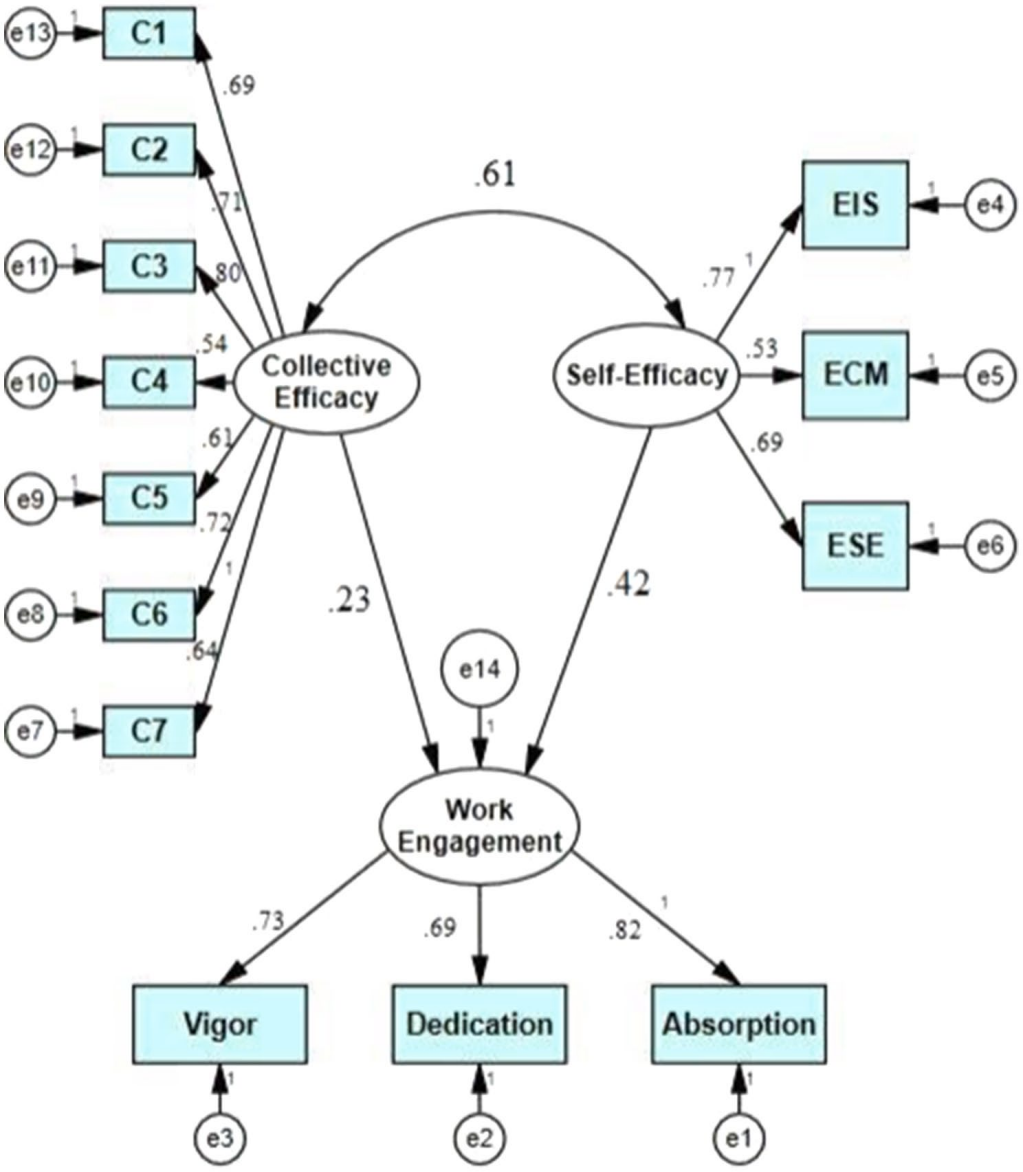
The model of the interplay among teacher collective efficacy (TSE), teacher self-efficacy (TSE), and WE.

Finally, to respond to the second research question, SEM was employed through Amos 24. For the strengths of the causal relationships among the components to be checked, the causal interplays among the factors were checked, using the analysis of the standardized evaluations. Figure 1 indicates the model of the interrelationships among TSE, TCE, and WE.

As indicated in Figure 1, both TCE (*β*=0.23, *p* <0.05) and TSE (*β*=0.42, *p*<0.05) are positive significant predictors of their WE. Finally, TCE correlated positively and significantly with TSE (*β*=0.61, *p*<0.05).

For the model fit to be checked, goodness of fit indices was utilized. Goodness of fit indices can be visible in Table 8. In this study, *χ*^2^/*df*, GFI, CFI, and RMSEA were employed. In order to have a fit model, χ^2^/*df* is required to be less than 3; GFI, CFI, and NFI are required to be above 0.90; and RMSEA is required to be less than 0.08.

**Table 8:** Goodness of fit indices.

	χ^2^/*df*	GFI	CFI	NFI	RMSEA
Acceptable fit	<3	>0.90	>0.90	>0.90	<0.08
Model	2.66	0.92	0.90	0.91	0.07

Table 8 delineates that all the integrity of fit indices can run inside the satisfactory level. Thus, the model had a reasonable level of validity.

## Discussion

The current study aimed to test a predictive role of TCE and TSE in WE among Chinese EFL teachers. Some crucial findings were put forward through this research. First, as Table 2 shows, the sig value for all the variables is higher than 0.05. Consequently, it can be summarized that there is a normal distribution across all three variables. As shown in Table 3, among the variables, WE has the highest mean (*M*=90.10, *SD*=11.06) while TCE obtained the lowest mean (*M*=26.35, *S*=4.77). Table 4 demonstrates the correlation among three variables in this study. As it can be implied, the highest correlation is ascribed to TCE and TSE (*r*=0.68, *p*=0.000). The second highest correlation can be seen between WE and self-efficacy (*r*=0.58, *p*=0.000). The third highest correlation was obtained between WE and TCE. Table 5 demonstrates that the relationship between all the sub-constructs of WE is positive and out of which dedication reached the highest (*r*=0.53, *p*=0.000). Considering all the subfactors in both TSE and WE, it can be perceived out of Table 6 that all the sub-constructs of WE, including vigor, dedication, and the absorption, are positively correlated with the sub-constructs of TSE, including “instructional strategies, classroom management, and student engagement,” among which the highest relationship can be found between instructional strategies and vigor. It implies that when a teacher has physical and mental energy and is also strong-willed, he is more likely to implement educational materials, strategies, and programs. It can also be understood that classroom management has the highest relationship with dedication. In other words, the more dedicated a teacher is, the better he can manage the classroom. Last but not least, the highest relationship between student engagement and vigor can be identified. To put it simply, the more determined and the more mentally and physically energetic a teacher is, the more engaged students can get. This finding is somewhat consistent with the following findings: approximately a forth of novice teachers do not continue working in their third year and about a third give up their profession when it has been just 5years from the moment they started teaching (Gold, 1996; Harris and Associates Inc., 1999). Studies have recommended that those who give up teaching are less efficacious than teachers continuing teaching (Glickman and Tamashiro, 1982). TSE has been said to be associated with stress that is experienced in teaching (Smylie, 1988). It is also in line with the findings of a study conducted by Greenier et al. (2021) that showed a significant positive correlation between emotion regulation and psychological wellbeing among a group of Iranian and British English teachers. It has also been found that there is a crucial link between TSE and emotion regulation (Fathi et al., 2021).

## Conclusion

One of the reasons behind this study is that more attention should be paid to teachers themselves, not just students since the striking role of teachers in learners’ achievements is extraordinarily crucial. Many studies were conducted to investigate the predictive role of TSE and TCE in students’ engagement despite the fact that in this study, efforts have been made to find the interplay between the following variables: TSE, TCE, and WE. WE comprises three sub-constructs, vigor which is characterized by how energetic and resilient a teacher is as working, the willingness to make an effort that is invested in working, and consistency when facing difficulties. Dedication is the second sub-construct of WE which refers to the level of involvement while working and gaining experience about a sense of importance, passion, inspiration, pride, and challenge. The third sub-construct of WE is absorption that refers to the full concentration and immersion when working (Maslach et al., 2001).

Consequently, all the afore-mentioned factors of WE should be boosted in teachers in order for them to experience a relaxing atmosphere in their jobs. Regarding all the three variables in this study, it has been shown that the more efficacious a teacher is, the more engaged he is in his work; it simply means that he is more determined, dedicated, persistent, energetic, inspired, and enthusiastic to do his job.

This study is, nevertheless, limited to some extent. First, instead of an experimental study, avid researchers can conduct a longitudinal study through which a teacher’s behavior would fully be analyzed, and then, the solution can be put forward so as to change teacher’s way of thinking that causes a specific behavior in their way of teaching.

Secondly, the participants of this study were chosen out of some provinces in China, while it has been proposed that one’s culture and region affects his teaching method; therefore, some other countries with different teachers can be the focus of future studies. That is, those participants from the cross-cultural contexts should be further investigated to generalize the research findings of the present study. Thirdly, from an institutional point of view, it is not enough to just conduct some studies regarding these issues which are of paramount significance; these studies should be implemented in the learning contexts to help teachers to build both TSE and TCE causing the main effect on their WE. Steps should be taken by the authorities to allow teachers to gain respect and feel more valued by providing them with the situation in which teachers are assisted to increase TSE. In this regard, attention will be drawn to teachers who are viewed as the most radical pillar in the learning contexts.

Further studies can be conducted in the future as well. A suggestion by which avid researchers can be intrigued is the main role of TSE and TCE on the extent to which a teacher can develop a sense of flexibility or be well-adjusted in both his personal life and work life. Those well-adjusted teachers are found to encourage the students to reach their apex in the learning contexts. As for students, teachers themselves can take advantage of being emotionally healthy in their own lives since the more stable a person is, the wiser decisions can be made by him which have a significant role in their way of teaching. Being well-adjusted is also in line with the level of serenity that can be experienced by teachers. Therefore, it could be another recommendation for future studies. Lastly, as has been suggested by one of my previous studies concerning research methodology along positive psychology that longitudinal and cross-cultural studies can be conducted to find the dynamic interplay of the variables which are subsumed under the umbrella of positive psychology movement in foreign and second language education (Yang, 2021).

## Data Availability Statement

The original contributions presented in the study are included in the article/supplementary material; further inquiries can be directed to the corresponding author.

## Ethics Statement

The studies involving human participants were reviewed and approved by the Ethics and Academic Committee of Henan University. The patients/participants provided their written informed consent to participate in this study.

## Author Contributions

The author confirms being the sole contributor of this work and has approved it for publication.

## Funding

This article was sponsored by the Academic Affairs Office of Henan University. The research project is entitled “The Exploration and Practice of Implementation of Moral Education in College English Follow-on Courses: A Case Study of Exploring the Charm of Yellow River Civilization (grant no. HDXJJG2020-05).”

## Conflict of Interest

The author declares that the research was conducted in the absence of any commercial or financial relationships that could be construed as a potential conflict of interest.

## Publisher’s Note

All claims expressed in this article are solely those of the authors and do not necessarily represent those of their affiliated organizations, or those of the publisher, the editors and the reviewers. Any product that may be evaluated in this article, or claim that may be made by its manufacturer, is not guaranteed or endorsed by the publisher.
